# A Wireless Electronic Nose System Using a Fe_2_O_3_ Gas Sensing Array and Least Squares Support Vector Regression

**DOI:** 10.3390/s110100485

**Published:** 2011-01-05

**Authors:** Kai Song, Qi Wang, Qi Liu, Hongquan Zhang, Yingguo Cheng

**Affiliations:** 1 School of Electrical Engineering and Automation, Harbin Institute of Technology, Harbin 150001, China; E-Mails: wangqi@hit.edu.cn (Q.W.); shf67@126.com (Q.L.); 2 Biochemistry Center, No.49 Institute of China Electronics Technology Group Corporation, Harbin 150001, China; E-Mails:zhanghq1@126.com (H.Z.); yyoogg.green@163.com (Y.C.)

**Keywords:** wireless electronic nose, combustible gas detection, Fe_2_O_3_ gas sensor, humidity insensitivity, DSP, least square support vector regression

## Abstract

This paper describes the design and implementation of a wireless electronic nose (WEN) system which can online detect the combustible gases methane and hydrogen (CH_4_/H_2_) and estimate their concentrations, either singly or in mixtures. The system is composed of two wireless sensor nodes—a slave node and a master node. The former comprises a Fe_2_O_3_ gas sensing array for the combustible gas detection, a digital signal processor (DSP) system for real-time sampling and processing the sensor array data and a wireless transceiver unit (WTU) by which the detection results can be transmitted to the master node connected with a computer. A type of Fe_2_O_3_ gas sensor insensitive to humidity is developed for resistance to environmental influences. A threshold-based least square support vector regression (LS-SVR)estimator is implemented on a DSP for classification and concentration measurements. Experimental results confirm that LS-SVR produces higher accuracy compared with artificial neural networks (ANNs) and a faster convergence rate than the standard support vector regression (SVR). The designed WEN system effectively achieves gas mixture analysis in a real-time process.

## 1. Introduction

An electronic nose (EN) is an instrument comprising diverse chemical sensors and an appropriate pattern recognition algorithm for detecting simple or complex odors [1,2]. As an innovative gas detection technology, EN systems combine sensor, electronics, signal processing and computer technology and are widely applied in many fields, e.g., medical diagnosis, food surveillance, production safety, environmental protection, *etc.* [3–6]. Most EN instruments employ a PC to control the data acquisition card and are described as desktop systems that are suitable for laboratory purposes [7,8]. Additionally, gas classification and concentration estimation are performed in two different processes [8,9]. More recent WEN systems combine chemical sensors with wireless sensor networks and are used to monitor the target gases via a remote system [10] or transmit the sensor array measurement data to a PC via wireless sensor nodes [9]. However, a real-time WEN instrument can not only acquire and transmit the sensor data by the RF transceiver but also process the data on-line through an embedded microcontroller as well as simultaneously transmit the gas species and concentration information to a desktop PC for intelligent management and human-computer interaction [10,11]. In addition, there is an urgent need for the development of high-performance WEN instruments that can detect on-line industrial leakage of gases within regulation ranges such as the explosion limits and threshold limit values [10]. To our knowledge, such real-time WEN systems for accurately quantifying complex combustible gas concentrations have rarely been reported [9–11].

Most reported EN systems used for industrial monitoring are based on metal oxide semiconductor (MOS) gas sensors [12,13]. MOS sensors, such as SnO_2_ [14], are a class of chemical sensors based on resistance changes. With their advantages of low cost, short response times, and high sensitivity to combustible gases, liquefied petroleum gas and organic solvent vapors, MOS sensors have become the most commercially suitable sensors in EN. However, MOS sensors have some well-known disadvantages, e.g., poor selectivity, cross-sensitivity, and the strong dependence on the external environment [15]. Inevitably, MOS sensors are sensitive to water vapor, which may be a problem for real-time monitoring in an industrial environment under variable humidity conditions [16]. Several promising approaches have been presented for improving the selectivity of MOS sensors by varying the category and percentage of additives [17,18]. Hardware and software methods have been adopted to address the problem of humidity compensation in EN systems [19–21]. For example, the ordered mesoporous SnO_2_ is insensitive towards changes in the relative humidity at low concentrations of carbon monoxide [22]. Fe_2_O_3_ sensors show great potential for industrial process monitoring, due to their fast response, high stability, high sensitivity [23] and especially their remarkably strong insensitivity to humidity [24–26].

The multivariable data processing techniques of EN systems can essentially be divided into two categories: statistical techniques and neural network techniques. The most important representatives of the former are principal component analysis (PCA) and multiple regression analysis, while ANNs fall into the latter. PCA is a linear feature extraction technique which is used to classify different odors [27,28] and multiple regression analysis is commonly employed as a quantitative measurement method for multicomponent mixtures. Recently, multiple linear regression (MLR), principal component regression (PCR) and partial least square regression (PLSR) have been successfully used in estimating concentrations of complex gas mixtures [20,29]. However, since MOS gas sensors commonly have a nonlinear characteristic, the methods mentioned above that were originally developed as linear regression methods will be invalid in a nonlinear model. ANNs use biologically inspired neural constructs and are similar to the human cognitive process. Detailed descriptions of ANNs applied in pattern recognition and quantitative analysis of the complex odors can be found in [30,31]. However, it may be difficult for ANNs to select hidden layers and the number of hidden units so that errors can occur when quantifications are not divided in detail during the training process. Inevitably, a large amount of network units will increase the computational complexity and require more training samples [32].

**Figure 1.**
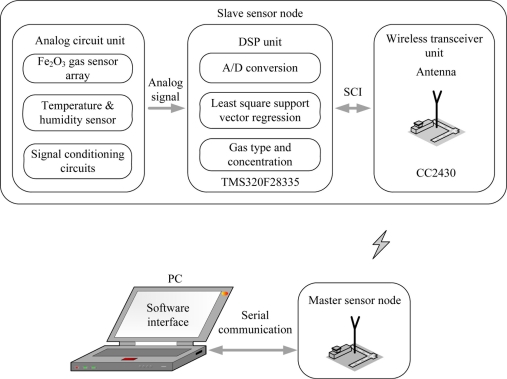
Block diagram of the WEN system.

A more attractive and effective pattern recognition method, support vector machine (SVM), is a set of supervised learning machine methods based on statistical learning theory [33]. SVM, which uses the principle of structural risk minimization (SRM), enhances the generalization ability and has been successfully applied to multidimensional EN data [8,34] with good classification and regression ability in the case of insufficient training samples [35]. Recently, least squares support vector machine (LS-SVM), an extension of standard SVM, was presented by Suykens [36]. LS-SVM regards a least square linear system as a loss function instead of the quadratic programming problem of the standard SVM, which simplifies operations, accelerates the convergence rate and improves the precision of the classification and nonlinear regression [37]. In this paper, a binary gas mixture analysis is regarded as a multivariate nonlinear regression problem. Classification and concentration estimation is synchronously fulfilled in a process. Meanwhile, the algorithm for quantifying gas mixtures, *i.e.*, the LS-SVR concentration estimator, is implemented in the DSP system (slave node) which can communicate with a desktop PC via a wireless sensor network, as shown in Figure 1.

This paper is organized as follows. Section 2 describes production engineering and performance test of the fabricated Fe_2_O_3_ gas sensors with high sensitivity and little humidity effects. In addition, temperature compensation circuits based upon thermistors are designed. Section 3 discusses the principle and algorithm flow of LS-SVR. Section 4 presents the hardware and software design of this WEN system. Section 5 is devoted to the experimental description and the principle of classification and concentration measurement by using the same LS-SVR model. Furthermore, discussion and comparison on the performance of the system for detection of the target odors (CH_4_, H_2_ and their mixtures) are provided. Finally, conclusions are drawn in Section 6.

## 2. Fabrication and Property Measurement of Fe_2_O_3_ Sensor

A Fe_2_O_3_ sensor is a semiconductor gas sensing device that can detect odors through the body resistance change. When the gas sensing slurry is fabricated, a sinter with a strong adhesion is simply shaped in the metal heater wire. Sensitivity and selectivity to different combustible gases can be changed by adding doping elements as well as controlling the grain size and the micro-structure of the sinter [26,38].

### 2.1. Gas Sensing Mechanism

Because of the deviation from stoichiometry and its active chemical nature (*i.e.*, easily reduced), the crystal defects of the gas sensing device are easily changed, which results in its body resistance change in contact with odors [25]. For example, a reduction reaction occurs when the gas sensing device contacts odors and Fe^2^+ ions are generated and changed into Fe_3_O_4_ with the increase of the gas concentrations so that body resistance of the device decreases [39]. At the same time, this change is reversible, as shown in Equation (1). The device will resume its original state when the testing gas is removed. Therefore, gas detection is achieved through such a reversible chemical transformation:

(1) $$\alpha -{\text{Fe}}_{2}O_{3}\overset{\text{Reduction}}{\underset{\text{Oxidation}}{⇄}}{\text{Fe}}_{3}O_{4}$$

The device will resume its original state when the testing gas is removed. Therefore, gas detection is achieved through such a reversible chemical transformation.

### 2.2. Sensor Fabrication

**Figure 2.**
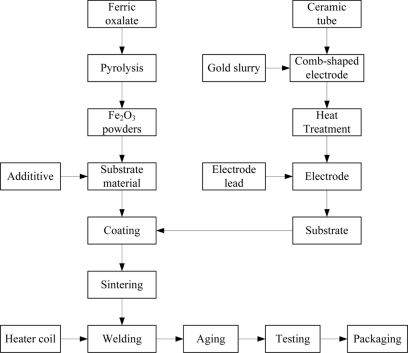
Fabrication diagram of the sintered Fe_2_O_3_ gas sensing device.

The Fe_2_O_3_ gas sensor fabrication process included fabrication of gas sensing material, additive selection, electrode preparation, substrate coating and sintering, lead welding, aging, packaging, *etc.*, as shown in Figure 2. In this paper, the chemical precipitation method was used to fabricate the α-Fe_2_O_3_ powders [38,40]. Firstly, 150 g ferrous sulfate [FeSO_4_·7H_2_O] were dissolved in 2,000 mL solution and the concentration of Fe^2+^ was 0.25 mol/L. Then 100 g Na_2_C_2_O_4_·2H_2_O was added to the solution:

(2) $$\begin{array}{c} {\text{FeSO}}_{4}\cdot 7H_{2}O+{\text{Na}}_{2}C_{2}O_{4}\cdot {2H}_{2}O\overset{\text{pH}=4,\;60{}^{\circ}C}{\to }{\text{FeC}}_{2}O_{4}\cdot 2H_{2}O↓+{\text{Na}}_{2}{\text{SO}}_{4}+{7H}_{2}O\\ 4{\text{FeC}}_{2}O_{4}\cdot {2H}_{2}O+{3O}_{2}\overset{820{}^{\circ}C,\;0.5h}{\to }2\alpha \text{-}{\text{Fe}}_{2}O_{3}+{8H}_{2}O↑+{8\text{CO}}_{2}↑ \end{array}$$

The whole procedure was carried out at 60 °C and the solution was adjusted to pH 4 in the reaction process. The precipitate was formed at the end of the reaction when the solution was cooled. Subsequently, the precipitate was centrifuged and washed with the distilled water, then the filtrate was dried at 80 °C and calcined at 820 °C for half an hour to thus form the α-Fe_2_O_3_ powders.

The prepared gas sensors used in this WEN system should have different sensitivity to the combustible gases in order to ensure the validity of multivariate analysis algorithms [16]. To adjust the selectivity of the sensors, heavy metals dopants were added to the powders. Experimental results show that the sensor has a greater sensitivity to CH_4_ when the content of SnO_2_ is 1.0–1.8% and the sensor has a greater sensitivity to H_2_ when the content of Sb is 0.7–1.6%. Therefore, sensors with different sensitivity to H_2_ and CH_4_ can be obtained by changing the content of additives.

The detailed process of electrode preparation, substrate coating, substrate sintering, lead welding, aging and packaging are described as follows: firstly, the prepared gold slurry was coated on the surface of the selected cylindrical ceramic tube in order to form a comb-shaped electrode. The coated ceramic tube was dried at room temperature and then calcined in the tube furnace. The temperature of the tube furnace was increased to 800 °C for 10 min and then dropped to room temperature. Electrode preparation could be accomplished after a diameter of 0.2 mm Pt wire as the electrode lead was welded to the calcined electrode. Secondly, the substrate material was ground into a paste and evenly coated on the ceramic tube. After natural drying, the substrate was calcined at 650 °C for 1 h. Subsequently, the calcined substrate was formed into small cylinders (diameter 1.5 mm × length 3 mm) where a heater wire could be put. Thirdly, a small Ni-Cr alloy wire (diameter 0.08 mm) used as the heater coil was placed on the ceramic tube and then the electrode lead and heater wire were welded to the base of the sensor. In order to improve the stability and the repeatability of the gas sensing device, the fabricated elements were placed in an aging platform and kept at 300 °C for 10 days in air. After aging, the characteristic parameters of each element were measured and the qualified ones were selected. Here, the elements were filtered according to resistances of sensors in the sample gas (H_2_ 1,000 ppm) and the resistance range was 100 K–1 M. By filtering, the qualified rate of a batch of elements were 73%. Finally, 100 mesh double stainless steel mesh was used to packet the element to complete fabrication of the Fe_2_O_3_ gas sensor.

### 2.3. Property Measurement

**Figure 3.**
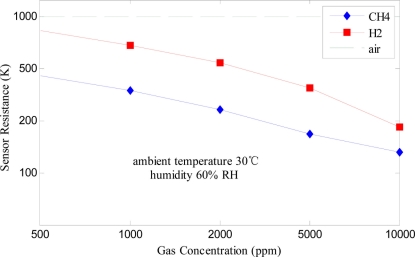
Sensitivity characteristics of the Fe_2_O_3_ gas sensor.

To detect leakage of combustible gases in the industrial field, stable sensors with high sensitivity, fast response and low dependence on the environmental conditions are required. Here, performance testing (sensitivity, response and recovery time, temperature and humidity characteristics, stability and life characteristic, *etc.*) was undertaken for the designed Fe_2_O_3_ gas sensor. Sensitivity curves of the sensor for the target odors (CH_4_ and H_2_) at 30 °C (ambient temperature) and 60% RH (relative humidity) are shown in Figure 3. It is obvious that the sensor’s resistance reduces while the combustible gas concentration increases and their relationship are nonlinear. Moreover, the sensor has high sensitivity to the two odors.

The response and recovery time of the gas sensor towards target odors are very important factors which relate directly to the rate of gas detection. We define response time t_res_ as the time to reach 90% the steady-state value of the sensor to the target odors and recovery time t_rec_ as the time to return back to 90% baseline in air. Table 1 shows the response and recovery time of the designed Fe_2_O_3_ gas sensor towards 2,000 ppm CH_4_ and H_2_ at room temperature, respectively. It can be seen from Table 1 that the response time is less than 10 s and recovery time is less than 30 s. Note that the response time and recovery time are not fixed values, which are affected by the gas type, concentration, operating temperature and airflow. In fact, many MOS sensors such as SnO_2_ are sensitive towards the ambient temperature and humidity. Sometimes changes in resistance of the sensor caused by the environment even exceed that caused by gas concentrations, which makes gas detection more complicated and inaccurate [15,16].

**Figure 4.**
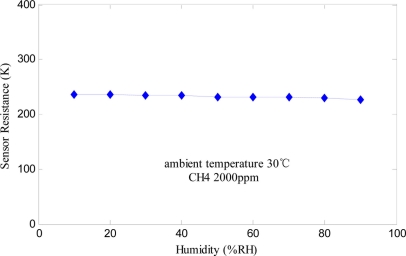
Humidity characteristic of Fe_2_O_3_ gas sensor.

To eliminate the influence of the ambient temperature and humidity, one way is to maintain the same experimental conditions for different data samples. This method is feasible for the constant temperature and humidity measurement chamber in the laboratory, but does not have any practical engineering value. In practice, hardware compensation circuits [19] or software compensation algorithms [21] are usually used for temperature and humidity compensation, and special sensors with humidity or temperature independency are thus developed. Figure 4 shows the humidity characteristic of the fabricated Fe_2_O_3_ gas sensor at 2,000 ppm CH_4_.

**Figure 5.**
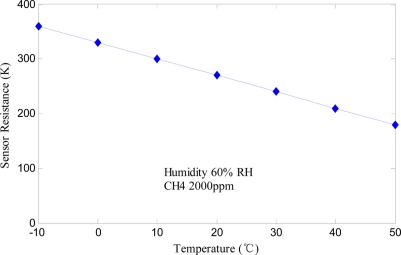
Temperature characteristics of the Fe_2_O_3_ gas sensor.

It is observed that sensor’s resistance remains the same when relative humidity ranges from 10% to 90%. Therefore, the Fe_2_O_3_ gas sensor is insensitive to humidity. When used as alarm instruments in industry, Fe_2_O_3_ gas sensing devices do not generate false positives due to the influence of water vapor. As shown in Figure 5, the sensor has a negative temperature coefficient and its resistance decreases with the temperature rise. Because of the humidity insensitivity of the Fe_2_O_3_ gas sensor, only temperature compensation needs to be done via a thermistor determined by the expected ambient temperature range. The principle of bridge compensation is that gas sensor’s resistance changes when the ambient temperature changes, and the thermistor resistance changes in the same direction [19]. In detail, the sensitivity characteristic curves of sensor in the usual condition of 20 °C and the extreme conditions of −10 °C and 40 °C were measured. The average curve was approximated at the above range of ambient temperature conditions and the temperature coefficient was calculated to decide the thermistor. From the above discussion, the α-Fe_2_O_3_ sensor developed in this paper, which has the advantages of rapid response velocity, high sensitivity to combustible gases, strong humidity insensitivity and long use life, is a promising type of gas sensor for industrial monitoring.

## 3. Least Squares Support Vector Regression

As previously mentioned, traditional multivariate data processing methods such as ANNs generally require enough training samples to ensure the generalization accuracy. This problem can be solved by LS-SVM, which shows excellent performance in solving small samples, nonlinear and local optimal points [37]. As reported in [36], LS-SVM adheres to the principle of SRM by minimizing an upper bound of the generalization error rather than minimizing the training error followed by ANNs. Particularly, it uses squares as the optimization index and substitutes equality constraints for inequality constraints of standard SVM so that the quadratic programming problem is converted into a linear equation, which reduces the computational complexity and improves the solution velocity. With Vapnik’s ε-insensitive loss function theory, SVM has been extended to solve the nonlinear regression problem known as SVR. When LS-SVM is used for regression, it equals LS-SVR. The basic idea of LS-SVR is that the input sample of data space is mapped into a higher dimensional feature space via a nonlinear mapping process, and then linear regression is obtained in feature space in order to indirectly accomplish the nonlinear regression in original space. A detailed description follows.

Consider a training sample set {(*x_k_*, *y_k_*)|*k* = 1,…,*N*} with input data *x_k_* ∈ *R^n^* and output *y_k_* ∈ *R*, where *N* denotes the number of training samples and *n* is the dimension of data space. In feature space ***H***, the LS-SVM model takes the form [36]:

(3) $$y(x)=w^{T}\phi (x)+b$$

where the nonlinear mapping φ(·) maps the input data into a higher dimensional feature space, *w* denotes the weight vector and *b* is a real constant namely the bias threshold [36]. In LS-SVR, the following optimization problem is formulated [37]:

(4) $$\text{min}\;J(w,e)=\frac{1}{2}w^{T}w+\frac{1}{2}\gamma \sum_{k=1}^{N}e_{k}^{2}$$

and subjected to:

(5) $$y_{k}=w^{T}\phi (x_{k})+b+e_{k},\;k=1,\;L,\;N$$

where the loss function *J* is the sum of SSE (sum of squared errors) and scale volumes, *γ* is a regularization parameter and *e_k_* are the error variables. Since LS-SVM has only the equality constraint conditions and loss function is the 2-norm of *e_k_*, the optimization problem can be greatly simplified. Define Lagrange function *L* as:

(6) $$L(w,b,e,\alpha )=J(w,e)-\sum_{k=1}^{N}\alpha_{k}\{w^{T}\phi (x_{k})+b+e_{k}-y_{k}\}$$

where *a_k_* are the Lagrange multipliers namely the support vectors. Exploit Karush-Kuhn-Tucher condition to optimize Equation (6) by:

(7) $$\left\{ \begin{array}{l} \frac{\partial L}{\partial w}=0\to w=\sum_{k=1}^{N}\alpha_{k}\phi (x_{k})\\ \frac{\partial L}{\partial b}=0\to \sum_{k=1}^{N}\alpha_{k}=0\\ \frac{\partial L}{{\partial e}_{k}}=0\to \alpha_{k}=\gamma e_{k}\\ \frac{\partial L}{{\partial \alpha }_{k}}=0\to w^{T}\phi (x_{k})+b+e_{k}-y_{k}=0 \end{array}\right.$$

For *k* = 1,…,*N*, substitute *e_k_* and *w* to obtain a matrix equation:

(8) $$\left[\begin{array}{cc} 0 & l^{T}\\ l & \Omega +\frac{1}{\gamma }I \end{array}\right]\;\left[\begin{array}{c} b\\ \alpha \end{array}\right]=\left[\begin{array}{c} 0\\ y \end{array}\right]$$

where *y* = [*y_1_*,…, *y_N_*]*^T^*, ***ι*** = [1,…, 1]*^T^*, *α* = [*α_i_*,…, *α_N_*]*^T^*, ***Ω****_kj_* = *φ*(*x_k_*)*^T^ φ*(*x_j_*),*j* = 1,…, *N*, ***I*** is a unit matrix and dimensions of ***l*** and ***I*** are *N*. Thus, optimization problem is transformed into the solution of linear equations. Note that *K*(·,·)is a kernel function that fulfills Mercer’s condition:

(9) $$K(x_{k},x_{j})=\phi {\left(x_{k}\right)}^{T}\phi (x_{j})\;k,\;j=1,\cdots ,N$$

Finally, the LS-SVR model for function estimator is described as:

(10) $$y(x)=\sum_{k=1}^{N}\alpha_{k}K(x,x_{k})+b$$

where *α_k_* and *b* can be solved by Equation (8). Here, the key issue is how to select an appropriate kernel function *K*(·,·) instead of the specific form of the nonlinear mapping *φ*. Some of the most widely used kernel functions include the radial basis kernel, polynomial kernel, sigmoid kernel and linear kernel, *etc.* The polynomial kernel and radial basis kernel always satisfy Mercer’s theorem, whereas other kernels satisfy it only for certain conditions [41]. In practice, the most commonly adopted one is the radial basis kernel function, namely:

(11) $$K\left(x_{i},x_{j}\right)=\text{exp}\left(-\frac{{\left\|x_{i}-x_{j}\right\|}^{2}}{\sigma^{2}}\right)$$

where the kernel parameter σ is specified *a priori*.

Above is the detailed LS-SVR calculation process. In this paper, the LS-SVR parameters were trained in advance via large numbers of experimental data from repeated measurements of the target gases. This training process was accomplished using Matlab language on a PC. After the regression model was trained, the related parameters were programmed in DSP and the real-time measurement data from the sensors could be used for the on-line analysis of unknown gas components.

## 4. Design and Implementation of the WEN System

### 4.1. Hardware Design

As shown in Figure 1, the slave node consists of a three parts-analog circuit unit, DSP unit and wireless transceiver unit. In the analog circuit, the gas sensor array composed of four of the developed Fe_2_O_3_ sensors is used to measure the target odors *i.e.*, CH_4_, H_2_ and their mixtures.

A temperature and humidity module is used to monitor the ambient temperature and relative humidity. Through the signal conditioning circuits, voltage signal outputs of the sensors are filtered and amplified, and then are acquired by the DSP for later data analysis. The TMS320F28335 is a high-performance 32-bit floating-point DSP whose working frequency is up to 150 MHz [42]. Additionally, it has a built-in 16-channel and 12-bit analog to digital converter (ADC) whose programmable acquisition rate throughput is up to 12.5 MSPS. It can synchronously acquire the voltage signal outputs of six sensors by the 32-bit timer as well as transfers data by direct memory access (DMA) without CPU, which greatly improves the velocity of data transmission. Real-time detection results are transmitted to the WTU (CC2430 module) by the serial communication interface (SCI) of the DSP. The low power wireless single chip, CC2430, which integrates a 2.4 GHz IEEE 802.15.4 compliant RF transceiver and an enhanced 8051 microcontrol unit (MCU), can achieve the wireless data transmission between the two nodes [43].

**Figure 6.**
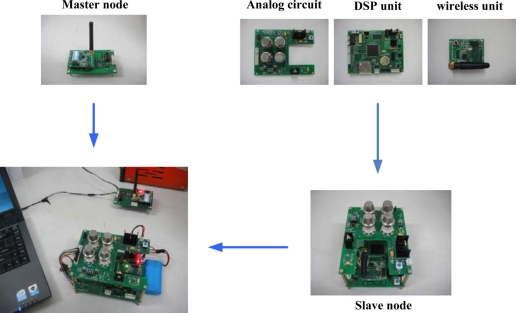
Photographs of the WEN system including slave node, master node and PC.

The main sensor node not only sends the detection results received from the slave node to PC but also receives the control instructions from PC and sends them to the slave node for controlling the DSP’s run. Figure 6 shows photographs of the designed WEN system including a slave node, a master node and a desktop PC.

### 4.2. Software Development

According to the hardware architecture of the designed WEN system, the tasks of the whole system are to achieve qualitative and quantitative detection of the combustible gases, wireless data transmission and information component display on a PC. Therefore, software development of the system includes three parts—DSP software design, WTU software design and PC software design.

**Figure 7.**
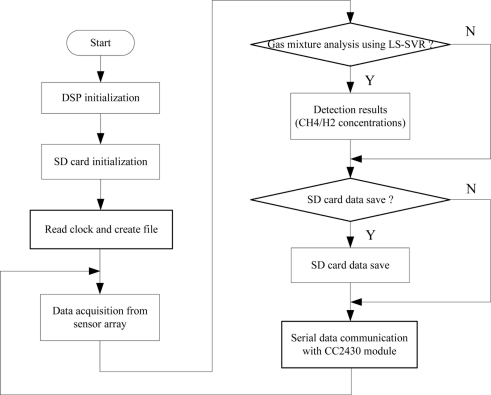
Flow diagram of the DSP program.

The DSP software design is the most important part of system software development. The DSP programs are designed in three steps. Firstly, the data acquisition program acquires the sensor array response data. Secondly, the LS-SVR multivariate analysis algorithm detects components of the analyte and then a SD card saves sensor array response data and detection results in the “txt” format. Finally, the serial communication program transmits the detection results to WTU. The specific program flow diagram is shown in Figure 7.

**Figure 8.**
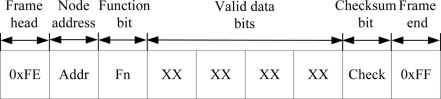
Wireless communication protocol.

**Figure 9.**
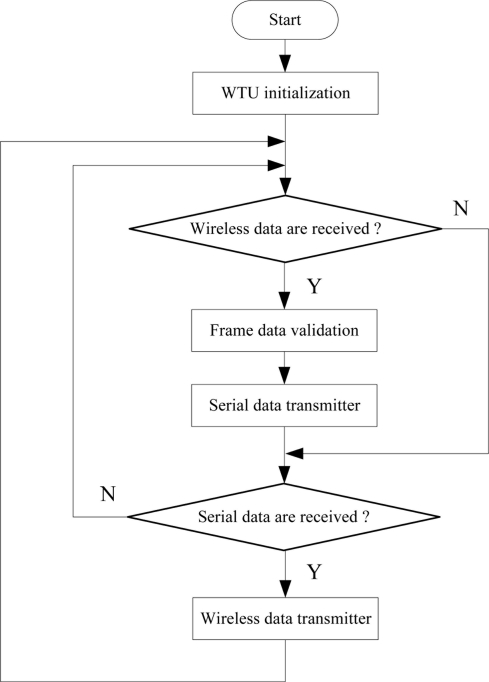
Flow diagram of the WTU program.

The WTU program is developed and compiled using the IAR Embedded Workbench software. The communication program of each node includes two parts: wireless communication with the other node and serial communication with the DSP (for slave node) or PC (for master node). The CC2430 wireless single chip integrates a wireless transceiver circuit in its internals, which provides the necessary hardware conditions for wireless communication. Therefore, we only program a wireless receiver and wireless transmitter function to achieve wireless data transmission between the two nodes. In addition, we design a wireless communication protocol and use the universal asynchronous receiver/transmitter (UART) interfaces of CC2430 to carry out the serial data transmission between WTU and DSP or PC. The data frame format is composed of frame head (0xFE), node address (Addr), function bit (Fn), four valid data bits, checksum bit (Check) and frame end (0xFF), as shown in Figure 8. Communication baud rate is set at 19,200 baud. The flow diagram of the communication program of nodes is shown in Figure 9.

The PC Software of the WEN system programmed in C++ language executes the gas detection result display and other related operations. The desktop PC is connected with the master node via a serial port which can achieve full-duplex serial communication. Functions of the software include the monitoring of the target gas concentrations, environmental temperature and humidity, working time and date, *etc.*

## 5. Experimental Results and Discussion

In our experiments, methane, hydrogen and their mixtures of different concentrations were chosen as the target odors. To verify the effectiveness of the developed WEN system, training and validation experiments were conducted. The former provided the samples to set up the LS-SVR model on the PC while the latter achieved the real-time gas component analysis by using the built model programmed in the designed WEN system.

### 5.1. Experimental Description

The experimental system based on the static gas distribution method [35,44]consisted of an organic glass gas chamber, a fan, two commercially available gas cylinders (99.99% CH_4_ and 99.99% H_2_), two syringes and two airbags. The slave node was powered via 7.4 V lithium batteries and was placed in the gas chamber whose effective volume was 10,000 mL. Four developed Fe_2_O_3_ sensors with different sensitivities to CH_4_ and H_2_ were installed in the slave node to form a sensor array. The heater voltage (V_H_) applied to the heater in order to maintain the sensor at 270 °C which is optimal for sensing was 5 V. The circuit voltage (V_C_) applied to allow voltage measurement across a load resistor which is connected in series with the sensor was 3.3 V. Temperature coefficient, *β* of the thermistor in the temperature compensation circuit was −5 × 10^−3^/°C. The master node connected with a desktop PC was used to fulfill the wireless data transmission with the slave node via the RF transceiver. CH_4_ and H_2_ were taken through the syringes from the airbags and injected into the gas chamber both singly and in mixtures. Different concentrations of the two gases and their mixtures could be made up by changing the amounts injected into the chamber. The fan in the chamber was used to aid the dispersal of the target odors. To illustrate the gas preparation process, we take the preparation of 1,000 ppm CH_4_ as an example. Since the volume of test container used in the experiments is 10,000 mL and the required concentration is 1,000 ppm (1,000/10^6^ = 10 mL/10,000 mL), only 10 mL of pure CH_4_ will be injected into the gas chamber with a 10 mL syringe. According to the lower alarm threshold limit (LATL) of the two combustible gases, *i.e.*, 20% of the lower explosion limit (LEL) and the responses of four sensors to their mixtures, the experimental concentration range of CH_4_ was 0–7,000 ppm and the concentration range of H_2_ was 0–5,000 ppm. When the baseline voltage in air was stabilized, the target odors were brought into the gas chamber and the responses were measured. The response curves were displayed on the monitor and the steady-state response voltages (V_O_) were recorded.

In the training experiments, the gas chamber temperature was maintained at 30 °C with 60% RH. The training samples of the analyte concentrations were shown in Table 2. Each measurement cycle was replicated 10 times for 19 training samples from those in Table 2, resulting in 190 measurements. As a result, the sensor array response made a 190 × 4 data set (*i.e.*, *N* = 190 samples and *n* = 4 sensors). Then the data set was processed using the Matlab language on the PC in order to build the LS-SVR model. We could thus compute the regression parameters via Equations (4–8) and obtain the concentration estimation results in Equation (10).

**Figure 10.**
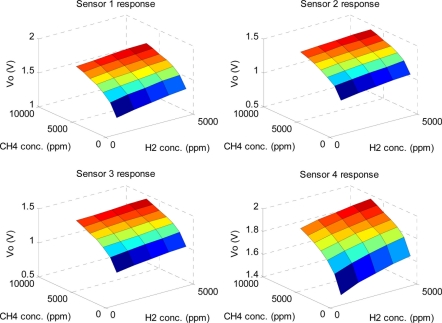
The average steady-state response distribution of the sensor array for the three target odors (CH_4_, H_2_ and their mixtures) in the training experiments.

Figure 10 shows the average steady-state response distribution of the sensor array for the three types of odors with different concentrations. It is apparent that the relationship between responses of individual sensors and gas concentrations is nonlinear, and the response of each sensor is a binary function of two gas concentrations. Here, the mean of each sensor’s response for concentration distribution in Figure 10 is calculated with 10 measured voltages, V_O_.

### 5.2. Results and Discussion

From the previous discussion, the procedures of applying LS-SVR for gas component analysis are summarized as follows:

1. Acquire gas concentrations and sensor array response as training samples.
2. Determine kernel function *K*(·,·) as well as the kernel parameters *σ* and *γ*.
3. Calculate regression parameters *α_k_* and *b* via Equation (8).
4. Establish the concentration estimator given in Equation (10).

The inputs of the regression model are the sensor array response and the outputs are the concentrations of the analyte both alone and in mixtures. Training samples are used to determine the kernel, calculate kernel parameters and establish the regression model. According to the physical characteristics of the Fe_2_O_3_ gas sensor, the radial basis kernel is more appropriate to capture the nonlinearity of the considered system compared with other kernels. Therefore, the radial basis kernel is adopted as the kernel function in this paper. The regularization parameter *γ* and kernel parameter *σ* is tuned experimentally by the *k*-fold cross-validation technique [45], where the training samples are randomly split into *k* approximately equal subsets. For each parameter set {*σ*, *γ*}, we train LS-SVR using *k*−1 subsets and check the generalization error using the subset left out. This procedure is repeated *k* times and in this fashion each subset is tested once. Averaging the testing error over the *k* trials, an estimation of the expected generalization error of the chosen parameter set {*σ*, *γ*} is derived. In this paper, the optimal parameters are selected as *σ*^2^ = 32, *γ* = 3,000 by minimizing the cross-validation error over the all parameter sets, then the regression parameters *α_k_* and *b* can be calculated by Equation (8).

The remaining task is to fulfill gas classification and concentration estimation with the built LS-SVR model which contains two groups of regression parameters (one for CH_4_ and the other for H_2_) programmed on DSP. Since the trained LS-SVR model outputs the estimated concentrations of target odors (CH_4_, H_2_ and their mixtures) quantitatively, a minimum threshold is used to judge whether each component exists or not in order to perform classification. Because of the direct impact on the gas classification accuracy, the threshold for CH_4_ and H_2_ is chosen as 200 ppm (0.5% LEL) according to the distinguishing rate of the system for the target odors. If the predicted concentration of the analyzed component is less than the threshold, it can be concluded that the component does not exist. For example, if the estimated gas concentrations are 47 ppm for CH_4_ and 3,991 ppm for H_2_, the qualitative classification result is that the analyte is only H_2_ but not CH_4_, and the quantitative measurement result is 0 ppm CH_4_ and 3,991 ppm H_2_. Therefore, types and concentrations of both single and complex gas can be measured in a synchronous calculation process.

**Figure 11.**
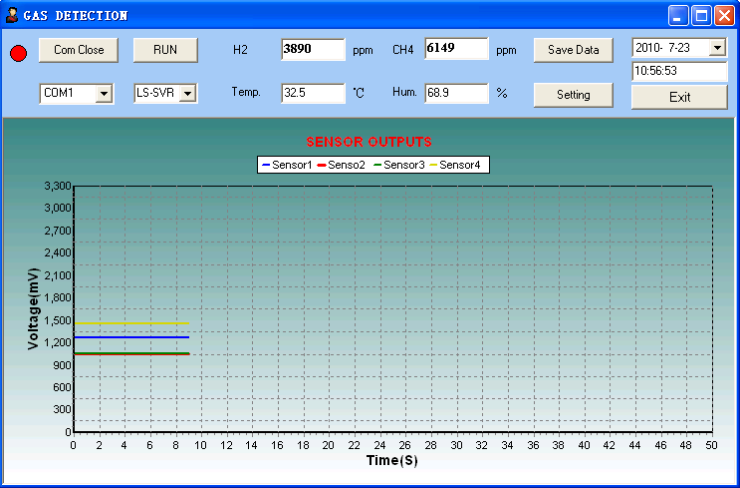
On-line measurement result of the WEN system for the arbitrarily selected analyte.

An important property that verifies performance of the system is its potential capability of predicting the out of range unknown analytes under different experimental conditions by using the in range training samples. Therefore, validation experiments were executed with arbitrarily selected analytes in order to test the accuracy, the repeatability and the real-time performance of the system for gas classification and concentration measurement. The 11 testing samples for validation are shown in Table 3. Since water vapor and atmosphere temperature are variables in a real environment, their influence on the response of gas sensor used for the system is supposed to be considered in the validation experiments. Figure 11 illustrates the on-line measurement result of the WEN system to the selected analyte (the mixture of 6,000 ppm CH_4_/4,000 ppm H_2_) in the condition of 32.5 °C and 70% RH. The four colored curves displayed on the PC software interface represent the steady-state response voltages of the four developed Fe_2_O_3_ sensors, respectively. The analyte was injected into the gas chamber 15 s later, and the component analysis result of 6,149 ppm CH_4_/3,890 ppm H_2_ was obtained from the LS-SVR concentration estimator. This demonstrates the fact that the designed WEN system can predict online the concentrations of the target odors with high precision under variable humidity conditions.

Table 3 reports the quantitative concentration measurement results and absolute errors analysis using this LS-SVR method in the validation experiments. It is apparent that the classification success rate is 100%. According to the absolute errors provided in Table 3, the worst-case prediction error is 8.8% for CH_4_ and is 12.8% for H_2_, and the average error of all testing samples is 2.9% for CH_4_ and 3.7% for H_2_. To further prove the performance of LS-SVR, the quantitative concentration measurement results using standard SVR and back-propagation artificial neural networks (BP-ANNs) for the same testing samples are given in Tables 4 and 5, respectively. The optimal regularization parameter *C* of SVR was chosen as 1,500 by minimizing the *k*-fold cross-validation error over the parameter sets [45]. The neural network consisted of an input layer with four nodes (*i.e.*, the sensor array response), a hidden layer with 12 nodes, and an output layer with two nodes which represented different components of the two gases, respectively. The initial weights for the neurons were chosen randomly, a hyperbolic tangent sigmoid function was used as the transfer function of the input layer to the hidden layer and a linear function was selected as the transfer function of the hidden layer to the output layer. The variable learning rate method [32] was used as the learning algorithm of the network.

Consider the correlation coefficient (*C.C*) (Table 6) as the index of estimation accuracy to evaluate the performance of the multivariable data processing methods [8]. *C.C* is a number between 0 and 1. The higher the correlation coefficient, the better the regression performance of the method. If the estimated concentrations are identical with the actual values, the correlation coefficient is 1. *C.C* is calculated as follows:

(12) $$C.C=\frac{\sum_{i=1}^{n}X_{i}{\hat{X}}_{i}-\frac{\sum_{i=1}^{n}X_{i}\sum_{i=1}^{n}{\hat{X}}_{i}}{n}}{\sqrt{\left(\sum_{i=1}^{n}X_{i}^{2}-\frac{{\left(\sum_{i=1}^{n}X_{i}\right)}^{2}}{n}\right)\left(\sum_{i=1}^{n}{\hat{X}}_{i}^{2}–\frac{{\left(\sum_{i=1}^{n}{\hat{X}}_{i}\right)}^{2}}{n}\right)}}$$

where *X* are the actual concentrations, *X̂* are the estimated concentration, and *n* is the number of testing samples.

Obviously, compared with BP-ANNs, LS-SVR applied to gas mixture analysis is capable of improving the accuracy of concentration measurements. This is because that the training samples (concentration interval for each target gas) are insufficient for ANNs to perform a precise estimation. It is unrealistic to get enough training samples of concentration distribution with a small interval, which will greatly increase the experimental complexity and time consumption of the learning process. Compared with standard SVR, LS-SVR performs higher accuracy for CH_4_ whereas it produces lower accuracy for H_2_. Furthermore, LS-SVR has a faster convergence rate than the standard SVR in the training process (Table 6). The reason is that LS-SVM solves the linear equations instead of the quadratic programming of the standard SVR. Linear equations have faster solution velocity and require fewer computing resources compared to quadratic programming.

In addition, traditional methods are needed to set up two different model architectures for gas component analysis. One is used as qualitative classification. The other served as a quantitative concentration measurement. However, the threshold-based method devised for the WEN system synchronously achieves qualitative and quantitative gas component analysis with only one built LS-SVR model, which dramatically reduces the computational complexity of DSP and can be suitably applied to a real-time electronic nose test system.

## 6. Conclusions

This paper develops a Fe_2_O_3_ gas sensor array based WEN system for detection of the main combustible gases CH_4_, H_2_ and their mixtures in industry. A type of Fe_2_O_3_ gas sensor with rapid response rate and high sensitivity to combustible gases was fabricated. This type of gas sensing element composed of a Ni-Cr integrated heater, and α-Fe_2_O_3_ powders that have remarkably strong insensitivity against humidity is particularly suitable for a real atmosphere environment under variable humidity conditions. A multivariable data processing method, LS-SVR, is presented to identify and quantify the binary analytes in a synchronous process. Compared with SVR and ANNs, the LS-SVR method devised for the WEN system requires less time and provides better concentration quantification of the analytes both singly and in mixtures.

## Figures and Tables

**Table 1.** Response and recovery time of Fe_2_O_3_ gas sensor at 2,000 ppm CH_4_ and H_2_.

**Time (s)**	**CH_4_**	**H_2_**
**t**_res_	5	9
**t**_rec_	15	29

**Table 2.** Concentration ranges of target odors for training. The symbol (√)in the table denotes the selected training samples.

**H_2_ (ppm)**	**CH_4_ (ppm)**				
	**0**	**1,000**	**3,000**	**5,000**	**7,000**
**0**		√	√	√	√
**1,000**	√	√	√	√	√
**3,000**	√	√	√	√	√
**5,000**	√	√	√	√	√

**Table 3.** Experimental results for validation using LS-SVR.

**Testing samples**	**Actual conc. (ppm)**		**Estimated conc. (ppm)**		**Absolute error (%)**	

	**CH_4_**	**H_2_**	**CH_4_**	**H_2_**	**CH_4_**	**H_2_**
1	2,000	0	2,176	0	8.8	0.0
2	4,000	0	3,928	0	1.8	0.0
3	6,000	0	5,736	0	4.4	0.0
4	0	2,000	0	2,091	0.0	4.6
5	0	4,000	0	3,991	0.0	0.2
6	2,000	2,000	2,089	2,255	4.5	12.8
7	2,000	4,000	1,979	4,308	1.1	7.7
8	4,000	2,000	4,141	2,017	3.5	0.9
9	4,000	4,000	4,232	3,673	5.8	8.2
10	6,000	2,000	5,996	2,077	0.1	3.9
11	6,000	4,000	6,149	3,890	2.5	2.8

**Table 4.** Experimental results of quantitative measurement using standard SVR.

**Testing samples**	**Actual conc. (ppm)**		**Estimated conc. (ppm)**		**Absolute error (%)**	

	**CH_4_**	**H_2_**	**CH_4_**	**H_2_**	**CH_4_**	**H_2_**
1	2,000	0	2,150	0	7.5	0.0
2	4,000	0	3,921	0	2.0	0.0
3	6,000	0	5,846	0	2.6	0.0
4	0	2,000	0	1,888	0.0	5.6
5	0	4,000	0	3,927	0.0	1.8
6	2,000	2,000	2,037	2,361	1.9	18.1
7	2,000	4,000	1,865	4,426	6.8	10.7
8	4,000	2,000	4,046	2,026	1.2	1.3
9	4,000	4,000	4,115	3,726	2.9	6.9
10	6,000	2,000	6,033	2,197	0.6	9.9
11	6,000	4,000	6,132	3,987	2.2	0.3

**Table 5.** Experimental results of quantitative measurement using BP-ANNs.

**Testing samples**	**Actual conc. (ppm)**		**Estimated conc. (ppm)**		**Absolute error (%)**	

	**CH_4_**	**H_2_**	**CH_4_**	**H_2_**	**CH_4_**	**H_2_**
1	2,000	0	2,310	0	15.5	0.0
2	4,000	0	3,831	0	4.2	0.0
3	6,000	0	5,814	0	3.1	0.0
4	0	2,000	0	2,059	0.0	3.0
5	0	4,000	0	4,111	0.0	2.8
6	2,000	2,000	2,105	2,396	5.3	19.8
7	2,000	4,000	1,933	4,352	3.4	8.8
8	4,000	2,000	4,036	2,192	0.9	9.6
9	4,000	4,000	4,224	3,787	5.6	5.3
10	6,000	2,000	6,053	2,116	0.9	5.8
11	6,000	4,000	6,303	3,931	5.1	1.7

**Table 6.** Performance evaluation of the three methods.

**Correlation coefficient**	**LS-SVR**	**SVR**	**BP-ANNs**
CH_4_	0.9981	0.9990	0.9972
H_2_	0.9948	0.9925	0.9944

**Training time (s)**	0.0238	12.8440	115.9940
